# CBCT-Based Assessment of External Apical Root Resorption in Clear Aligner Versus Fixed Orthodontic Therapy: A Systematic Review and Meta-Analysis

**DOI:** 10.3390/healthcare14111547

**Published:** 2026-06-02

**Authors:** Ersin Yıldırım, Celalettin Topbas

**Affiliations:** 1 Department of Orthodontics, Hamidiye Faculty of Dentistry, University of Health Sciences, Istanbul 34668, Türkiye; 2 Department of Endodontics, Hamidiye Faculty of Dentistry, University of Health Sciences, Istanbul 34668, Türkiye; celalettin.topbas@sbu.edu.tr

**Keywords:** external apical root resorption, orthodontic tooth movement, clear aligners, fixed orthodontic appliances, cone-beam computed tomography, orthodontics, root length, meta-analysis

## Abstract

**Background/Objectives**: External apical root resorption (EARR) is a recognized biological consequence of orthodontic tooth movement. Although clear aligner therapy has been hypothesized to reduce EARR due to staged and intermittent force delivery, existing evidence remains heterogeneous, particularly with respect to imaging modality. This systematic review and meta-analysis aimed to compare EARR between clear aligners and fixed orthodontic appliances using exclusively quantitative cone-beam computed tomography (CBCT)-based linear root length measurements. **Methods**: A systematic search was conducted in PubMed/MEDLINE, Scopus, Web of Science, Embase, CENTRAL, ClinicalTrials.gov, WHO ICTRP, and Google Scholar (inception to January 2026). Comparative clinical studies reporting CBCT-based linear root length changes (mm) in clear aligner and fixed appliance groups were included. Random-effects meta-analysis (DerSimonian–Laird method) was performed to calculate pooled mean differences (MD = CA − FA). Heterogeneity was assessed using Cochran’s Q and I^2^ statistics. Risk of bias was evaluated using the ROBINS-I tool, and certainty of evidence was assessed with GRADE. **Results**: Six studies involving 392 patients met the inclusion criteria and were included in the quantitative synthesis. Random-effects meta-analysis demonstrated significantly lower EARR in the clear aligner group compared with fixed appliances (MD = −0.50 mm; 95% CI −0.79 to −0.21; *p* < 0.001). Between-study heterogeneity was moderate (I^2^ = 60.8%). Subgroup analysis suggested a larger reduction in extraction-based protocols, although data were limited. Sensitivity analyses confirmed the robustness of the overall effect. The certainty of evidence was rated as low. **Conclusions**: Clear aligner therapy was associated with lower CBCT-measured external apical root resorption compared with fixed orthodontic appliances; however, this finding should be interpreted with caution due to the limited number of studies, moderate heterogeneity, and potential residual confounding inherent to non-randomized designs. The results should be considered exploratory rather than definitive.

## 1. Introduction

External apical root resorption (EARR) is a well-recognized iatrogenic consequence of orthodontic tooth movement and constitutes an important biological limitation of orthodontic treatment. Histopathologically, EARR involves irreversible loss of cementum and dentin at the root apex and may compromise tooth integrity and long-term prognosis, particularly when resorption becomes extensive. The classic biological and clinical frameworks describing orthodontically induced inflammatory root resorption emphasize that, although mild degrees of resorption are frequent and often clinically acceptable, severe EARR can adversely affect tooth longevity and remains a major concern for clinicians and patients alike [1,2].

The etiology of EARR is multifactorial and reflects a complex interaction between patient susceptibility and treatment-related mechanical loading. Patient-related factors such as age, root morphology, and genetic predisposition have been associated with variability in EARR risk [3,4,5]. Treatment-related factors—including force magnitude, force duration, force continuity, total treatment time, appliance system, and the extent or type of tooth movement—are also repeatedly implicated as determinants of both initiation and progression of EARR [6,7]. Mechanistically, orthodontic forces induce inflammatory and cellular responses within the periodontal ligament (PDL) and adjacent alveolar bone; sustained or excessive loading can promote hyalinization and osteoclastic activity at the root surface, thereby increasing resorption potential [8,9,10].

Among treatment-related variables, the orthodontic appliance system is clinically relevant because it shapes force delivery and movement control. Fixed orthodontic appliances are frequently associated with EARR—particularly in anterior teeth where tipping, torque, and intrusive mechanics may be required—while the wire–bracket interface may generate complex and relatively continuous force systems [11,12]. In contrast, clear aligner therapy has expanded rapidly due to esthetic demands and digital workflow advancements. Biomechanically, aligners typically apply staged, intermittent forces through sequential aligner changes, theoretically allowing periods of force relief and potentially reducing cumulative biological insult to the root–PDL complex [13,14,15]. These features have generated the clinical hypothesis that aligner-based treatment may be associated with less EARR than fixed appliances.

Clinical studies comparing EARR between clear aligners and fixed appliances have reported inconsistent findings, which is partly explained by methodological heterogeneity—especially regarding imaging modality and outcome definition [16,17,18,19]. Historically, EARR assessment has relied on two-dimensional (2D) imaging (panoramic or periapical radiographs), which is vulnerable to projection errors, distortion, and sensitivity to changes in tooth angulation, potentially underestimating root length changes [20,21]. Cone-beam computed tomography (CBCT) enables three-dimensional visualization and offers improved accuracy and reproducibility for quantitative root length evaluation relative to 2D radiography [22,23,24]. Accordingly, CBCT-based measurement is increasingly preferred in research settings when precise quantification of EARR is required.

Several systematic reviews, meta-analyses, and higher-level evidence syntheses have addressed EARR associated with aligners versus fixed appliances. However, an important recurring limitation is that many reviews have pooled heterogeneous outcome definitions and imaging methods (mixing CBCT with 2D measures), which can compromise comparability and quantitative interpretation [25,26,27]. A recent umbrella review further summarized the existing review-level evidence, reinforcing that conclusions remain constrained by heterogeneity of methods and primary studies [28]. Unlike previous reviews that combined heterogeneous imaging modalities, this study exclusively included CBCT-based quantitative measurements to reduce methodological heterogeneity and improve measurement accuracy.

Therefore, consistent with our PROSPERO-registered protocol, the objective of this study is to systematically review and meta-analyze clinical studies comparing clear aligner therapy versus fixed orthodontic appliances with EARR assessed using quantitative CBCT-based linear root length measurements in millimeters (mm). By restricting inclusion to CBCT-derived linear outcomes and applying updated comprehensive searches, this review aims to provide a methodologically robust and clinically interpretable comparison of EARR across these two widely used orthodontic treatment modalities.

## 2. Materials and Methods

### 2.1. Protocol Registration and Reporting Standards

This systematic review and meta-analysis was prospectively registered in the International Prospective Register of Systematic Reviews (PROSPERO; Registration No. CRD420261320269). The study was conducted and reported in accordance with the Preferred Reporting Items for Systematic Reviews and Meta-Analyses (PRISMA 2020) statement and its updated checklist [29].

A completed PRISMA 2020 checklist is provided in the Supplementary Materials (Supplementary Table S1). The study design, eligibility criteria, outcome definitions, and analytical approach strictly followed the registered protocol to ensure methodological transparency and to minimize selective reporting bias.

### 2.2. Eligibility Criteria (PICO and Outcome Definition)

Eligibility criteria were defined a priori according to the PICO framework:

Population (P): Human subjects undergoing orthodontic treatment.

Intervention (I): Clear aligner therapy (e.g., Invisalign or other thermoplastic aligner systems).

Comparator (C): Fixed orthodontic appliances (e.g., conventional brackets, self-ligating brackets, passive self-ligating systems).

Outcome (O): External apical root resorption (EARR) assessed using quantitative CBCT-based linear root length measurements (millimeters).

Studies were included if they:

- Compared clear aligner therapy with fixed orthodontic appliances.
- Used CBCT imaging for EARR assessment.
- Reported quantitative root length changes (mm) or provided sufficient data to calculate mean differences.
- Were randomized clinical trials (RCTs) or non-randomized clinical studies.

Studies were excluded if they:

- Used only two-dimensional radiographic assessment.
- Reported qualitative scoring without quantitative data.
- Were case reports, reviews, in vitro studies, animal studies, or finite element analyses.
- Did not provide a direct comparison between aligner and fixed appliance groups.

### 2.3. Information Sources and Search Strategy

A comprehensive electronic search was conducted from database inception to January 2026 in the following databases:

- PubMed/MEDLINE;
- Scopus;
- Web of Science Core Collection;
- Embase (via Ovid);
- Cochrane Central Register of Controlled Trials (CENTRAL).

To minimize publication bias, grey literature searches were performed in:

- ClinicalTrials.gov;
- WHO International Clinical Trials Registry Platform (ICTRP);
- Google Scholar (first 200 results sorted by relevance).

The search strategy combined controlled vocabulary and free-text terms related to:

- “external apical root resorption”;
- “clear aligner” OR “Invisalign”;
- “fixed orthodontic appliance” OR “braces” OR “brackets”;
- “cone beam computed tomography” OR “CBCT”.

The search strategy was initially developed for PubMed and subsequently adapted for each database according to its specific syntax and indexing system. The detailed search strategies for each database are provided in the Supplementary Materials (Supplementary Table S2).

No restrictions were applied regarding publication year. Only studies published in English were included due to resource limitations for translation.

### 2.4. Screening and Eligibility Assessment

All records were imported into reference management software and duplicates were removed. Two independent reviewers screened titles and abstracts for eligibility. Potentially relevant articles underwent full-text assessment.

Disagreements were resolved through discussion or consultation with a third reviewer.

**Figure 1:**
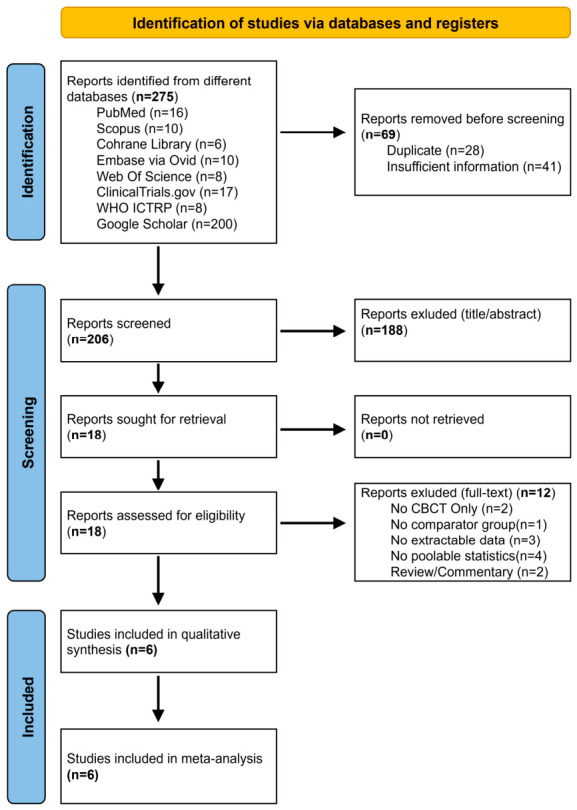
PRISMA 2020 flow diagram of study selection.

The study selection process is illustrated in the PRISMA flow diagram (Figure 1).

### 2.5. Data Extraction

Two reviewers independently extracted data using a standardized form. Extracted variables included:

- Author and year;
- Country;
- Study design;
- Sample size;
- Mean age and sex distribution;
- Extraction protocol (extraction vs. non-extraction);
- CBCT acquisition parameters (voxel size, field of view);
- Root length measurement protocol;
- Mean root length change (mm);
- Standard deviation (SD);
- Follow-up duration.

When necessary, corresponding authors were contacted to obtain missing or unclear data, in accordance with the registered protocol.

CBCT imaging in the included studies was performed based on clinical indications rather than solely for research purposes.

### 2.6. Assessment of Risk of Bias

Risk of bias was independently evaluated by two reviewers.

- Randomized controlled trials were assessed using the Cochrane Risk of Bias tool (RoB 2) [30].
- Non-randomized studies were assessed using the ROBINS-I tool [31].

Each domain was judged as low, moderate, serious, or critical risk of bias (for ROBINS-I) or low/some concerns/high risk (for RoB 2).

### 2.7. Effect Measure and Data Synthesis

The primary outcome was the mean difference (MD) in apical root length change (mm) between clear aligner and fixed appliance groups (CA − FA). Effect direction was defined as MD = CA − FA; negative values indicate less EARR with aligners. Unit of analysis was patient-level mean apical root length change. Data were extracted at the patient level where available to minimize unit-of-analysis bias.

When studies reported more than one fixed appliance arm, group means and standard deviations were combined using standard formulas for pooling independent groups prior to meta-analysis.

Meta-analysis was performed using a random-effects model (DerSimonian–Laird method), considering anticipated clinical and methodological heterogeneity across studies [32]. Between-study variance (tau-squared, τ^2^) was estimated using the DerSimonian–Laird estimator.

Heterogeneity was assessed using Cochran’s Q test and quantified using the I^2^ statistic. I^2^ values were interpreted as follows: 0–25% (low heterogeneity), 26–50% (moderate heterogeneity), 51–75% (substantial heterogeneity), and >75% (considerable heterogeneity).

The unit of analysis was the study-level mean root length change. When tooth-level data were reported, study-level averages were extracted to avoid unit-of-analysis errors.

Meta-analysis was performed in R (R Foundation for Statistical Computing, Vienna, Austria) using the metafor package with a random-effects model (DerSimonian–Laird estimator) [33].

Given the observational nature of the included studies, residual confounding related to treatment allocation and case complexity was anticipated and considered during interpretation of the results.

### 2.8. Subgroup and Sensitivity Analyses

Pre-specified subgroup analyses were conducted based on:

- Extraction vs. non-extraction protocols;
- Study design (RCT vs. non-randomized);
- Tooth type (anterior vs. mixed, when data permitted).

Sensitivity analyses were performed by excluding one study at a time to evaluate the robustness of pooled estimates.

### 2.9. Assessment of Reporting Bias

If ≥10 studies were available, publication bias was planned to be assessed using funnel plots and Egger’s regression test [34]. Given that fewer than 10 studies were included, formal statistical tests for publication bias (e.g., Egger’s regression test) are underpowered and may yield misleading results. Therefore, publication bias was assessed descriptively and interpreted with caution.

### 2.10. Certainty of Evidence

The certainty of evidence was evaluated using the GRADE framework [35], considering:

- Risk of bias;
- Inconsistency;
- Indirectness;
- Imprecision;
- Publication bias.

The overall certainty of evidence was classified as high, moderate, low, or very low.

## 3. Results

### 3.1. Study Selection

The literature search identified 275 records across databases and trial registries. After the removal of duplicates and records with insufficient information (*n* = 69), 206 records were screened. Following title and abstract screening, 188 records were excluded. Eighteen full-text articles were assessed for eligibility, of which 12 were excluded for predefined reasons (no CBCT-only assessment, *n* = 2; no comparator group, *n* = 1; no extractable data, *n* = 3; no poolable statistics, *n* = 4; review/commentary, *n* = 2).

Six studies met the inclusion criteria and were included in the qualitative and quantitative synthesis (k = 6) [16,17,18,36,37,38].

The study selection process is illustrated in Figure 1.

### 3.2. Study Characteristics

The characteristics of the included studies are summarized in Table 1.

The six studies comprised 392 patients and directly compared clear aligner (CA) therapy with fixed appliance (FA) treatment using CBCT-based linear root length measurements (mm) [16,17,18,36,37,38].

Three studies included mixed or heterogeneous treatment protocols (including extraction and non-extraction cases) [16,18], while extraction status was not clearly specified in one pilot study [36], two were non-extraction focused [17,37], and one specifically evaluated extraction-based Class II treatment [38]. All studies reported patient-level mean apical root shortening in millimeters suitable for quantitative synthesis.

### 3.3. Risk of Bias Assessment

Risk of bias was evaluated using ROBINS-I [31]. No randomized controlled trials met the inclusion criteria; therefore, RoB 2 was not applied. Domain-level and overall judgments are presented in Table 2.

Most studies were judged as having moderate risk of bias due to potential confounding inherent to non-randomized designs. One pilot study [36] was rated as serious risk of bias. No study was considered at critical risk.

### 3.4. Quantitative Synthesis (Primary Outcome: CBCT Linear EARR in mm)

Extracted CBCT-based linear root resorption data used for meta-analysis are shown in Table 3.

Using a DerSimonian–Laird random-effects model [32], clear aligner therapy demonstrated significantly lower EARR compared with fixed appliances:

Pooled MD = −0.50 mm (95% CI −0.79 to −0.21; *p* < 0.001).

Heterogeneity statistics are summarized in Table 4:

- Q = 12.76;
- df = 5;
- Tau^2^ = 0.077;
- I^2^ = 60.8%.

**Figure 2:**
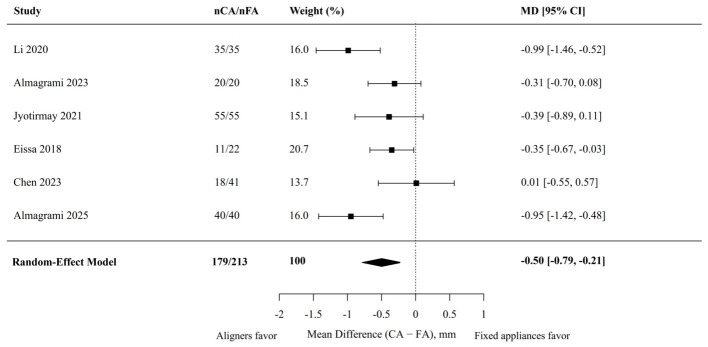
Forest plot of the overall meta-analysis including six studies [16,17,18,36,37,38]. Random-effects meta-analysis comparing the magnitude of orthodontically induced external apical root resorption (EARR) between clear aligners (CA) and fixed appliances (FA) assessed using cone-beam computed tomography (CBCT). Effect sizes are expressed as mean differences (MD) with 95% confidence intervals (CI). Negative values indicate lower EARR associated with clear aligner therapy. Squares represent study-specific estimates weighted by inverse variance and the diamond represents the pooled effect estimate.

The overall forest plot is presented in Figure 2.

The direction of effect favored aligners in five of the six studies [16,17,18,36,38], while one study [37] demonstrated a near-null difference.

### 3.5. Subgroup Analysis (Extraction Protocol)

**Figure 3:**
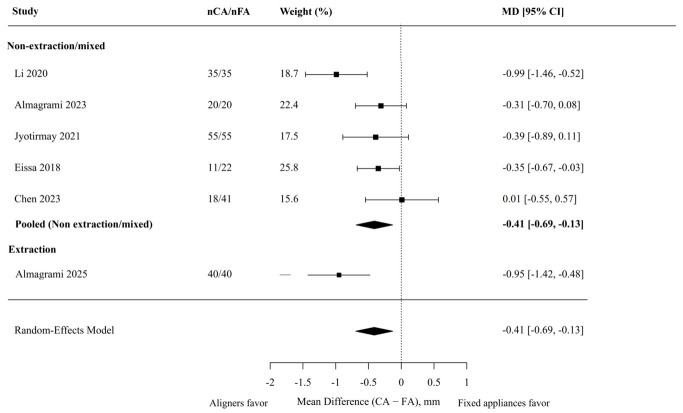
Subgroup analysis according to treatment protocol (non-extraction/mixed vs. extraction) [16,17,18,36,37,38]. A pooled estimate was calculated for the non-extraction/mixed subgroup, while the extraction subgroup contained a single study and therefore no pooled estimate was calculated.

Pre-specified subgroup analysis according to extraction protocol is presented in Table 5 and visualized in Figure 3.

- Non-extraction/mixed subgroup (k = 5) [16,17,18,36,37]:

MD = −0.41 mm (95% CI −0.69 to −0.13; I^2^ = 52.5%).

- Extraction subgroup (k = 1) [38]:

MD = −0.95 mm (95% CI −1.42 to −0.48).

The reduction in the root length remained statistically significant in the non-extraction subgroup. Non-extraction/mixed subgroup pooled estimates should be interpreted cautiously due to clinical heterogeneity.

The extraction subgroup included only one study; therefore, no statistical comparison or pooled inference can be made, and results are only presented descriptively.

### 3.6. Sensitivity Analysis

Sensitivity analyses excluding studies at higher risk of bias are summarized in Table 6. Sensitivity analyses were conducted by excluding the pilot study (serious ROBINS-I) and by excluding the extraction-only study to explore protocol-driven heterogeneity.

Exclusion of the study rated as serious risk [36] yielded:

MD = −0.53 mm (95% CI −0.81 to −0.25; I^2^ = 55%).

Exclusion of the extraction-only study [38] resulted in:

MD = −0.41 mm (95% CI −0.69 to −0.13; I^2^ = 52.5%).

Across all sensitivity scenarios, the pooled effect remained statistically significant.

### 3.7. Leave-One-Out Influence Analysis

Leave-one-out analysis results are presented in Table 7.

Sequential exclusion of each study did not alter the statistical significance or direction of the pooled effect. The pooled MD ranged from −0.41 mm to −0.60 mm depending on the study removed, indicating absence of dominant study influence.

### 3.8. Assessment of Reporting Bias

**Figure 4:**
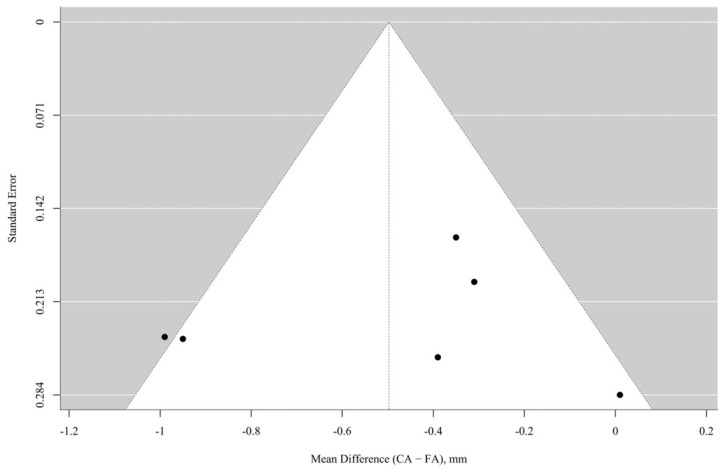
Funnel plot evaluating potential small-study effects and publication bias. Each point represents an individual study plotted according to its effect size and standard error.

The publication bias assessment is presented in Table 8, and the funnel plot is shown in Figure 4.

Egger’s regression test [34] showed:

- Intercept = −1.42;
- *p* = 0.28.

Egger’s regression test did not demonstrate statistically significant small-study effects (*p* = 0.28). However, given the small number of included studies (k = 6), this analysis is underpowered and should be interpreted with caution. Due to the limited number of studies, the funnel plot visualization is also presented for exploratory purposes only. Accordingly, no definitive conclusions regarding publication bias can be drawn from the present dataset.

### 3.9. Certainty of Evidence

Using the GRADE framework [35], the certainty of evidence for the primary outcome was rated as low due to:

- Observational study designs;
- Moderate heterogeneity;
- Limited number of included studies.

The GRADE Summary of Findings is presented in Table 9.

## 4. Discussion

This systematic review and meta-analysis synthesized the best available clinical evidence comparing external apical root resorption (EARR) between clear aligner therapy and fixed orthodontic appliances, restricted to studies using quantitative CBCT-based root length outcomes. By limiting inclusion to CBCT-derived linear measurements, we aimed to reduce measurement bias associated with two-dimensional radiographs and enhance cross-study comparability [20,21,22,23]. The principal finding is that clear aligner therapy is associated with less CBCT-measured apical root shortening than fixed appliances, although the certainty of evidence remains constrained by the non-randomized nature of most included studies and residual clinical heterogeneity [31,35]. However, the limited number of included studies restricts the robustness of the conclusions and reduces the ability to draw definitive clinical inferences.

Across six eligible clinical studies directly comparing aligners with fixed appliances using CBCT-based linear root length change (mm) [16,17,18,36,37,38], the pooled effect favored aligners, indicating a moderate reduction in apical root shortening relative to fixed appliances. While the mean difference is numerically modest, it should be interpreted in the context of typical orthodontic EARR magnitudes in anterior teeth and the cumulative biological burden of sustained force systems [6,7,11,12]. Importantly, severe EARR—although less frequent—has been associated with compromised root length and potential long-term prognosis concerns in susceptible teeth, especially maxillary incisors [1,2,24]. Accordingly, even moderate reductions in mean apical shortening may be clinically relevant for patients with higher baseline susceptibility (e.g., atypical root morphology, trauma history, or anticipated extensive movement) [3,4,5,24].

A key interpretive point is that EARR is not binary; rather, it exists along a spectrum in which small mean differences may reflect meaningful shifts in the tail of the distribution (i.e., fewer “high-resorption” cases). Because most primary studies report group means, the present synthesis primarily informs average effect and does not fully resolve whether aligners reduce the probability of severe EARR at the individual level—an important direction for future research.

The direction of effect is biologically plausible. Orthodontically induced inflammatory root resorption is mediated by inflammatory and cellular responses within the periodontal ligament (PDL) and adjacent bone, influenced by force magnitude, duration, and continuity [8,9,10]. Sustained forces can promote hyalinization and recruitment of clastic cells at the root surface, increasing resorptive activity [8,9,10]. Evidence from classic experimental work indicates that interrupted force application is associated with less root resorption than continuous force systems, supporting a mechanistic basis for differences between appliance modalities [7].

Fixed appliances frequently deliver relatively continuous forces through archwire engagement and continuous activation during alignment and space closure, particularly when torque, intrusive mechanics, and complex tooth movements are required [11,12]. In contrast, aligners commonly apply staged forces with intermittent “force-off” periods between aligner changes, potentially reducing cumulative PDL stress and the duration of sustained hyalinization [7,13,14,15]. Additionally, digital planning inherent to aligner therapy may facilitate more controlled movement trajectories in select scenarios, potentially limiting uncontrolled intrusive vectors or excessive tipping—mechanical patterns often implicated in higher EARR risk [6,7,11,12]. Nevertheless, aligners can also exhibit “uncontrolled tipping” under certain biomechanical conditions, and this limitation is clinically important when interpreting the findings and generalizing them to complex malocclusions.

Between-study heterogeneity was moderate, which is unsurprising given the clinical diversity in extraction protocols, malocclusion types, appliance systems (including passive self-ligating brackets), and CBCT acquisition parameters [16,17,18,36,37,38]. Treatment mechanics and the extent of movement are established determinants of EARR [6,7], and these may differ substantially across cohorts even within the same nominal “aligner” or “fixed” category.

Consistent with our protocol, extraction versus non-extraction (or mixed) treatment protocols represent a particularly plausible source of heterogeneity. These sources of variability substantially limit the clinical interpretability of the pooled estimate and reduce the ability to generalize findings across all orthodontic treatment scenarios. Extraction therapy generally involves larger sagittal tooth movement and space closure mechanics that may increase biological risk for EARR, especially in anterior teeth [6,7,11,12]. Therefore, the observation that extraction-focused data can show larger absolute resorption values in both arms, while still favoring aligners, should be interpreted as hypothesis-supporting rather than definitive when subgroup evidence is limited in size or number of studies. Although the extraction-based study also favored aligners, the evidence is insufficient to draw meaningful conclusions regarding extraction protocols.

Sensitivity analyses (e.g., leave-one-out) are essential in a synthesis with a small number of studies because pooled estimates can be disproportionately influenced by a single study with larger sample size, larger effect, or lower variance [32]. The stability of the direction of effect across most included studies supports a consistent association, but precision is still constrained by limited study count and between-study variability.

Publication bias and small-study effects are difficult to judge reliably with fewer than 10 studies. Although funnel plot visualization can be presented, it remains exploratory under these conditions, and formal statistical testing (e.g., Egger’s test) is underpowered and may be misleading [34]. Accordingly, any statements about publication bias should be cautious and framed as inconclusive rather than confirmatory.

A critical methodological nuance in orthodontic EARR research is the unit of analysis. Some studies report outcomes per patient (e.g., averaged across teeth), whereas others report per tooth or per incisor subgroup. It should be noted that primary studies did not consistently account for clustering effects (multiple teeth per patient), and intraclass correlation coefficients were not reported, which may have resulted in overestimation of precision. Therefore, pooled confidence intervals should be interpreted cautiously. Tooth-level analyses, if treated as independent without appropriate clustering adjustment, can inflate precision and narrow confidence intervals, leading to overconfident inferences. Where possible, meta-analyses should prefer patient-level summary measures or appropriately derived composite estimates. The present synthesis prioritized extractable group-level summaries aligned with the protocol outcome definition (CBCT-based linear root length change, mm), but future primary studies should explicitly model within-patient clustering and report both tooth-level and patient-level summaries.

Measurement heterogeneity also matters. Although CBCT offers superior accuracy and reproducibility over 2D methods for root length assessment [20,21,22,23], acquisition parameters (voxel size, field of view, reconstruction algorithms) and measurement protocols (landmark selection, observer calibration) can still influence estimates and contribute to heterogeneity. Standardization of CBCT protocols and reporting would improve comparability and reduce measurement-driven variability.

Even when the pooled estimate is statistically significant, the confidence in the effect should be considered through the lens of imprecision and information size. In GRADE terms, imprecision is evaluated by the width of the confidence interval and whether it includes clinically important benefit or harm [35]. Here, the interval favors aligners and does not cross the null, supporting a directionally consistent conclusion. However, because EARR lacks a universally accepted minimal clinically important difference threshold, “clinical importance” should be presented as contextual rather than absolute. This supports a cautious statement: aligner therapy is associated with less EARR on average, but the degree of clinical benefit may vary by patient susceptibility, mechanics, and treatment protocol.

Previous systematic reviews and meta-analyses have reported inconsistent conclusions regarding EARR differences between aligners and fixed appliances, largely due to heterogeneous imaging modalities and outcome definitions, including pooling CBCT measures with 2D radiographic outcomes [25,26,27]. Two-dimensional imaging is vulnerable to projection error and sensitivity to tooth angulation changes, which can under- or mis-estimate true root length changes and reduce validity in pooled quantitative comparisons [20,21,22,23]. A recent umbrella review similarly emphasized that the review-level conclusions remain constrained by heterogeneity of primary studies and methodological inconsistencies [28]. The present synthesis advances the field by focusing on a consistent, quantitative CBCT-based linear endpoint and incorporating updated searches, thereby improving interpretability while still acknowledging limitations driven by study design and heterogeneity.

From a clinical standpoint, these results suggest that aligner therapy may represent a potentially less biologically burdensome option in selected clinical scenarios with respect to apical root shortening for selected patients, particularly those at higher risk for EARR [3,4,5,24]. Nevertheless, appliance selection must remain individualized. Aligners have biomechanical limitations in certain complex movements, and uncontrolled tipping or insufficient root control may occur depending on staging, attachments, and compliance. Therefore, a reduction in mean EARR should not be interpreted as a universal protective effect across all malocclusions or mechanics; rather, it supports the need for risk stratification, careful biomechanical planning, and monitoring. Importantly, EARR is primarily driven by biomechanical factors and individual susceptibility rather than appliance type alone.

Early radiographic detection of EARR remains critical during orthodontic care, as early changes can progress if risk factors persist [24]. Where clinically justified, appropriate imaging and periodic reassessment may help guide force modulation, treatment pacing, or mechanics adjustments in susceptible patients.

The present meta-analysis demonstrated that treatment with clear aligners was associated with significantly lower external apical root resorption compared with fixed appliances (MD = −0.50 mm). Although the absolute magnitude of this difference may appear modest, it may be clinically relevant in susceptible patients, although the patient-level clinical importance remains uncertain. In such patients, treatment modalities that potentially reduce the biological burden on the periodontal ligament may be advantageous. Therefore, the present findings should not be interpreted as evidence that clear aligners universally prevent EARR or are inherently superior across all orthodontic scenarios.

The findings should be interpreted in light of several limitations. First, despite comprehensive searching, the number of eligible CBCT-linear comparative studies remains limited, restricting the ability to explore multiple sources of heterogeneity or perform robust meta-regression. Sources of heterogeneity include differences in treatment duration, type of tooth movement (e.g., intrusion, torque), aligner protocols (attachments, staging), and CBCT acquisition parameters. Second, most included studies were non-randomized, and residual confounding is likely even with careful clinical matching and risk of bias assessment using ROBINS-I [31]. Residual confounding due to treatment allocation and case complexity cannot be excluded and may partially explain the observed effect. Clear aligners are often used in less complex cases, which may introduce selection bias. Therefore, appliance-related differences cannot be fully separated from underlying differences in biomechanical complexity. Third, variation in extraction protocols, tooth types evaluated, and measurement parameters contributes to heterogeneity and limits generalizability across all orthodontic contexts. Fourth, publication bias assessment is inherently limited with a small number of studies; funnel plot patterns and Egger-type testing are not definitive under these conditions [34]. Fifth, inconsistent reporting of unit-of-analysis handling and CBCT acquisition parameters, measurement variability, including voxel size, landmark identification, and observer calibration further constrains interpretability and underscores the need for standardized reporting. Additionally, variability among aligner systems (material properties, thickness, wear protocols) and fixed appliance systems further limits direct comparability between studies. Differences in malocclusion severity, extent of tooth movement, previous orthodontic treatment, and dental trauma were not consistently reported and pooling these may limit clinical interpretability. Therefore, the pooled estimate should be interpreted as an overall trend rather than a uniform clinical effect.

Future studies should prioritize well-designed prospective comparative cohorts and, where feasible, randomized trials with standardized mechanics and CBCT acquisition protocols. Reporting should include voxel size, reconstruction details, reliability metrics, and explicit handling of clustering when tooth-level outcomes are analyzed. Beyond mean root length change, future work should report distributional outcomes (e.g., proportion exceeding clinically meaningful thresholds) and consider patient-level predictors to identify subgroups most likely to benefit. Consensus-driven CBCT-based EARR outcome definitions and core outcome sets would substantially strengthen future evidence synthesis.

## 5. Conclusions

Within the limitations of the currently available evidence, clear aligner therapy was associated with lower CBCT-measured external apical root resorption compared with fixed orthodontic appliances. However, the evidence base remains limited by non-randomized study designs, residual confounding, and substantial clinical heterogeneity. Therefore, these findings should be interpreted cautiously and considered hypothesis-supporting rather than clinically definitive. Further well-designed prospective studies are required.

## Figures and Tables

**Table 1:** Characteristics of included studies (quantitative synthesis, k = 6).

Study	Country	Design	n (CA/FA)	Malocclusion/Protocol	Extraction	Teeth Assessed	CBCT Outcome	Unit of Analysis
Li et al., 2020 [16]	China	Retrospective cohort	35/35	Mixed malocclusion	Mixed	Maxillary incisors	Linear root length change (mm)	Patient-level mean
Almagrami et al., 2023 [17]	Saudi Arabia	Comparative clinical	20/20	Non-extraction	No	Maxillary incisors	Linear root length change (mm)	Patient-level mean
Jyotirmay et al., 2021 [18]	India	Retrospective cohort	55/55	Mixed orthodontic cases	Mixed	Anterior teeth	Linear root length change (mm)	Patient-level mean
Eissa et al., 2018 [36]	Egypt	Pilot comparative	11/22	Class I	No	Maxillary incisors	Linear root length change (mm)	Patient-level mean
Chen et al., 2023 [37]	China	Comparative clinical	18/41	Class II Div 2	No	Maxillary central incisors	Linear root length change (mm)	Patient-level mean
Almagrami et al., 2025 [38]	Saudi Arabia	Comparative clinical	40/40	Class II Div 1	Yes	Maxillary incisors	Linear root length change (mm)	Patient-level mean

CA, clear aligner treatment; FA, fixed appliance treatment.

**Table 2:** ROBINS-I risk of bias assessment.

Study	Confounding	Selection of Participants	Classification of Intervention	Deviations from Intended Intervention	Missing Data	Measurement of Outcome	Selection of Reported Result	Overall Risk
Li 2020 [16]	Moderate	Low	Low	Low	Low	Low	Low	Moderate
Almagrami 2023 [17]	Moderate	Moderate	Low	Low	Low	Low	Low	Moderate
Jyotirmay 2021 [18]	Moderate	Low	Low	Low	Low	Low	Low	Moderate
Eissa 2018 [36]	Serious	Moderate	Low	Low	Moderate	Low	Low	Serious
Chen 2023 [37]	Moderate	Moderate	Low	Low	Low	Low	Low	Moderate
Almagrami 2025 [38]	Moderate	Low	Low	Low	Low	Low	Low	Moderate

All six included studies were non-randomized comparative clinical studies; ROBINS-I was applied [31]. No study was judged as critical risk. One pilot study (Eissa 2018) [36] was judged as serious risk due to small sample size, baseline imbalance risk, and limited control for confounding.

**Table 3:** Extracted CBCT data used for meta-analysis.

Study	n (CA)	Mean ± SD (CA) *	n (FA)	Mean ± SD (FA) *	MD (CA − FA)	95% CI **
Li 2020 [16]	35	0.13 ± 0.47	35	1.12 ± 1.34	−0.99	−1.46 to −0.52
Almagrami 2023 [17]	20	0.31 ± 0.42	20	0.62 ± 0.78	−0.31	−0.71 to 0.09
Jyotirmay 2021 [18]	55	1.12 ± 1.34	55	1.51 ± 1.34	−0.39	−0.89 to 0.11
Eissa 2018 [36]	11	0.44 ± 0.35	22	0.79 ± 0.59	−0.35	−0.65 to −0.05
Chen 2023 [37]	18	0.92 ± 0.87	41	0.91 ± 1.26	0.01	−0.52 to 0.54
Almagrami 2025 [38]	40	0.90 ± 0.97	40	1.85 ± 1.18	−0.95	−1.42 to −0.48

CBCT, cone-beam computed tomography; CA, clear aligner treatment; FA, fixed appliance treatment; SD, standard deviation; MD, mean differences; CI, confidence interval. * Outcome expressed as external apical root resorption (linear root length change) measured in millimeters using CBCT. ** Study-level 95% CIs were calculated from group means, SDs, and sample sizes assuming independent groups.

**Table 4:** Random-effects meta-analysis (primary outcome).

Parameter	Value
Pooled mean difference (MD)	−0.50 mm
95% confidence interval	−0.79 to −0.21 mm
Z value	3.39
*p* value	<0.001
Cochran’s Q	12.76
Degrees of freedom	5
Heterogeneity p	0.026
Tau^2^	0.077
I^2^	60.8%
Model	DerSimonian–Laird random-effects

**Table 5:** Pre-specified subgroup analysis (extraction protocol).

Subgroup	Studies (k)	Pooled MD (mm)	95% CI	I^2^
Non-extraction/mixed	5	−0.41	−0.69 to −0.13	52.5%
extraction	1	−0.95	−1.42 to −0.48	—

MD, mean differences; CI, confidence interval. Note: Extraction subgroup includes Almagrami 2025 [38] only; heterogeneity not estimable.

**Table 6:** Sensitivity analysis (high-risk study exclusion).

Scenario	Studies (k)	Pooled MD (mm)	95% CI	I^2^
Main analysis	6	−0.50	−0.79 to −0.21	60.8%
Excluding Eissa 2018 [36]	5	−0.53	−0.81 to −0.25	55%
Excluding Chen 2023 [37]	5	−0.60	−0.88 to −0.32	52%
Excluding Almagrami 2025 [38]	5	−0.41	−0.69 to −0.13	52.5%

MD, mean differences; CI, confidence interval. Interpretation: Effect remained statistically significant across all sensitivity scenarios → robustness confirmed.

**Table 7:** Leave-one-out influence analysis.

Study Removed	New Pooled MD (mm)	95% CI	I^2^
None (full model)	−0.50	−0.79 to −0.21	60.8%
Li 2020 [16]	−0.41	−0.70 to −0.12	58%
Almagrami 2023 [17]	−0.52	−0.81 to −0.23	61%
Jyotirmay 2021 [18]	−0.53	−0.82 to −0.24	59%
Eissa 2018 [36]	−0.53	−0.81 to −0.25	55%
Chen 2023 [37]	−0.60	−0.88 to −0.32	52%
Almagrami 2025 [38]	−0.41	−0.69 to −0.13	52.5%

MD, mean differences; CI, confidence interval. Interpretation: No single study altered direction or statistical significance → no dominant influence.

**Table 8:** Assessment of publication bias.

Parameter	Value
Intercept (Egger)	−1.42
Standard error	1.11
t value	−1.28
*p* value	0.28
Interpretation	No statistical evidence of small-study effects

Egger’s regression test was performed (k = 6; exploratory only) [34]. Funnel asymmetry interpretation is limited due to small number of studies (<10).

**Table 9:** GRADE summary of evidence.

Outcome	Participants	Effect (MD)	Certainty	Interpretation
External apical root resorption (mm)	392 (6 studies)	−0.50 mm (95% CI [−0.79 to −0.21])	Low	Aligners may be associated with lower mean apical root shortening; however, certainty of evidence is low.

Certainty of evidence is assessed using the GRADE [35] framework.

## Data Availability

No new data were created or analyzed in this study. Data sharing is not applicable to this article.

## Supplementary Materials

The following supporting information can be downloaded at https://www.mdpi.com/article/10.3390/healthcare14111547/s1, Table S1: PRISMA 2020 Checklist; Table S2: Detailed Database Search Strategy.
