# Supplementary material for: CBCT-Based Assessment of External Apical Root Resorption in Clear Aligner Versus Fixed Orthodontic Therapy: A Systematic Review and Meta-Analysis
Source: Healthcare (Basel). 2026 Jun 2;14(11):1547. doi: 10.3390/healthcare14111547 (PMC13256781; doi:10.3390/healthcare14111547)
Supplement: Supplementary file 1 [file healthcare-14-01547-s001.zip › Table_S1.pdf]

Supplementary Table S1. PRISMA 2020 Checklist

**Section**	**Item**	**Reported in Manuscript**
**Title**	Identify as systematic review/meta-analysis	Title page
**Abstract**	Structured summary	Abstract
**Introduction**	Rationale	Introduction
	Objectives	Introduction
**Methods**	Protocol & registration	Methods 2.1
	Eligibility criteria	Methods 2.2
	Information sources	Methods 2.3
	Search strategy	Table S2
	Study selection	Methods 2.4
	Data collection	Methods 2.5
	Risk of bias	Methods 2.6
	Effect measures	Methods 2.7
	Synthesis methods	Methods 2.7–2.8
	Reporting bias	Methods 2.9
	Certainty assessment	Methods 2.10
**Results**	Study selection	Figure 1
	Study characteristics	Table 1
	Risk of bias	Table 2
	Results of synthesis	Tables 3–9
**Discussion**	Summary of evidence	Discussion
	Limitations	Discussion
	Conclusions	Discussion, Conclusion
**Other**	Support statement	Declarations
	Competing interests	Declarations
	Availability of data	Declarations

Checklist prepared according to PRISMA 2020 reporting guidelines for systematic reviews and meta-analyses.
