# Supplementary material for: CBCT-Based Assessment of External Apical Root Resorption in Clear Aligner Versus Fixed Orthodontic Therapy: A Systematic Review and Meta-Analysis
Source: Healthcare (Basel). 2026 Jun 2;14(11):1547. doi: 10.3390/healthcare14111547 (PMC13256781; doi:10.3390/healthcare14111547)
Supplement: Supplementary file 1 [file healthcare-14-01547-s001.zip › Table_S2.pdf]

Supplementary Table S2. Detailed Database Search Strategy

Database	Search Strategy	Records Identified
PubMed / MEDLINE	("external apical root resorption" OR "root resorption") AND ("clear aligner*" OR "Invisalign") AND ("fixed orthodontic appliance*" OR "brackets" OR "braces") AND ("cone beam computed tomography" OR CBCT)	16
Scopus	TITLE-ABS-KEY ("external apical root resorption" OR "root resorption") AND TITLE-ABS-KEY ("clear aligner*" OR Invisalign) AND TITLE-ABS-KEY ("fixed orthodontic appliance*" OR brackets OR braces) AND TITLE-ABS-KEY ("cone beam computed tomography" OR CBCT)	10
Cochrane CENTRAL	("external apical root resorption" OR "root resorption") AND ("clear aligner*" OR Invisalign) AND ("fixed orthodontic appliance*" OR brackets OR braces) AND ("cone beam computed tomography" OR CBCT)	6
Embase (Ovid)	('external apical root resorption'/exp OR 'root resorption') AND ('clear aligner' OR invisalign) AND ('fixed orthodontic appliance' OR brackets OR braces) AND ('cone beam computed tomography' OR cbct)	10
Web of Science Core Collection	TS=("external apical root resorption" OR "root resorption") AND TS=("clear aligner*" OR Invisalign) AND TS=("fixed orthodontic appliance*" OR brackets OR braces) AND TS=("cone beam computed tomography" OR CBCT)	8
ClinicalTrials.gov	"fixed orthodontic appliance" AND "clear aligner" AND "root resorption" AND ("cone beam computed tomography" OR CBCT)	17
WHO ICTRP	"fixed orthodontic appliance" AND "clear aligner" AND "root resorption" AND ("cone beam computed tomography" OR CBCT)	8
Google Scholar	("fixed orthodontic appliance" OR brackets OR braces) AND ("clear aligner" OR Invisalign) AND "root resorption" AND ("cone beam computed tomography" OR CBCT)	200
**Total**		**275**

Searches were conducted from database inception to January 2026.

The Google Scholar search was restricted to the first 200 results sorted by relevance to identify potentially relevant grey literature.
